# Cancer treatment in the last 6 months of life: when inaction can outperform action

**DOI:** 10.3332/ecancer.2018.826

**Published:** 2018-04-18

**Authors:** Bishal Gyawali, Saroj Niraula

**Affiliations:** 1 Program on Regulation, Therapeutics and Law (PORTAL), Division of Pharmacoepidemiology and Pharmacoeconomics, Brigham and Women’s Hospital, Harvard Medical School, Boston, MA 02120, USA; 2 Department of Medical Oncology and Hematology, University of Manitoba and Cancer Care Manitoba, Winnipeg R2H 2A6, Canada

**Keywords:** end of life, financial toxicity, overall survival, serious adverse events, fatal adverse events

## Abstract

When an investigational anticancer drug is being tested, demonstration of improvement in overall survival (OS) will generally lead to regulatory approval. However, the value that improvement in OS adds to patients’ lives is guided largely by the context of the improvement and accompanying trade-offs. For example, when a patient’s life expectancy is less than 6 months, many oncologists will not embark on any active cancer treatments. However, multiple new anticancer drugs have been approved recently after being tested in end-stage cancer patients and demonstrating median OS in the experimental arm close to 6 months. Such practice, particularly when the treatment is also accompanied by serious toxicities and cost, can undermine a peaceful life-death transition. In this commentary, we review regulatory approvals in the last 5 years and the ethical considerations involved in testing active cancer treatment in terminal cancer patients.

Recently, a fully human IgG4 monoclonal antibody inhibitor of programmed death-1 (PD-1), nivolumab (Opdivo), was reported to show a statistically significant improvement in overall survival (OS) compared to placebo in terminal gastric or gastro-oesophageal junction cancers [1]. Although the 1 month improvement in survival observed in this trial is a real gain, the trial also showed that the median OS, regardless of the randomisation arm, was less than 6 months. Although formal measures of quality of life were not presented in the trial report, potentially debilitating toxicities, treatment-related deaths and drug discontinuations were higher in the experimental arm. Nivolumab is also a highly expensive drug.

Many oncologists will not recommend active cancer treatment when they recognise that their patients are in the last few months of life [2–4]. Cancer patients will have usually undergone a series of invasive treatments including surgery, radiotherapy and drug therapy during the course of their disease. These limited last few months are precious for cancer patients and their families, and when patients are on active therapy, it will lead to time being spent visiting treatment rooms and enduring toxic effects of cancer treatments. The financial toxicity of high-cost therapies administered during this time can be another source of stress for patients and their families.

## Why do we continue prescribing drugs in the last 6 months?

One obvious answer is that we do not know how best to predict survival—physicians are known to be particularly bad at this and they tend to be unrealistically optimistic [5]. Physicians and patients tend to have exaggerated confidence in the benefits of drugs regardless of the disease setting, despite many studies confirming the futility of chemotherapy in the last 6 months of life [3, 6, 7]. Furthermore, many patients receiving chemotherapy for incurable cancers do not realise that chemotherapy is unlikely to be curative, which can lead to increased desire for aggressive treatments and high threshold of acceptable toxicities [8]. Drug websites that patients use to seek information on cancer therapies can provide overestimates of drugs’ benefits and downplay their harms [9].

## Last 5 years of cancer drugs approval

After the publication of the ATTRACTION trial [1], we explored systematically the frequency of drug approvals when OS is less than 6 months. Since United States Food and Drug Administration (FDA) approval of a drug often signals that other regulators should approve the drug in their countries, we examined data available publicly on the FDA’s website [10] and checked survival data for cancer drugs approved for the treatment of advanced or metastatic solid cancers between January 2012 and December 2016. In this time period, there were a total of 70 drugs approved, including new indications for drugs already on the market. We found three cancer drugs (4.3%) approved during this timeframe that were tested in a population which had a median OS of 6.5 months or fewer with the drug (Table 1). This is likely an underestimation of drugs meeting this criteria, since patient survival in routine practice is inferior to that observed in the controlled setting of clinical trials [11].

The three drugs were regorafenib (Stivarga) for later line treatment of metastatic colorectal cancer, ramucirumab (Cyramza) for the second line treatment of metastatic gastric or gastro-oesophageal cancer and liposomal irinotecan (Onivyde) for the second line treatment of metastatic pancreatic cancer. Coincidentally, all these three drugs, as well as the trial of nivolumab in gastric cancer mentioned above, are for cancers of the gastrointestinal system. For the nivolumab example, it may well be argued that the median doesn’t adequately capture the benefit with PD-1 drugs [20]. However, the same cannot be argued for these three drugs, which are not immunotherapies.

The additional survival benefit stemming from the use of these drugs as compared to the control arm was less than 2 months (1.4–1.9 months). Thus, the trade-off for efficacy with these drugs observed in the trials was between 3.8 and 5.0 months of drug-free life *versus* 5.2–6.4 months of life with drugs. When these drugs are considered for use by cancer patients and their oncologists, these survival gains should be balanced against the potential clinical toxicities from these drugs, including the financial toxicities to the patient and family.

## How toxic are these agents?

Regorafenib, ramucirumab and liposomal irinotecan are associated with a variety of toxicities, including substantially increased risks of serious and fatal adverse events (FAEs). Patients who are in the last 6 months of their lives considering the use of these drugs should, therefore, be adequately informed that their anticancer agents could put them at risk for serious adverse events (SAEs) including death in the hope of prolonging life by less than 2 months at the median.

## How expensive are these agents?

Despite providing minimal benefit and being associated with various potentially fatal toxicities, these drugs cost more than $9500 every month in the US. These costs may be borne to differing degrees by the patients themselves, depending on their insurance status. Since many cancer patients are on public insurance programs in the US such as Medicare and Medicaid, it is also worth considering whether it is sustainable at the societal level to pay over $9500 every month for a drug that extends survival by less than 2 months for patients who are in the last 6 months of their lives. Resources are finite: spending in such low value drugs can lead to compromises in access to other life-saving treatments, or in valuable societal priorities such as childhood education [12, 13].

## Role of the media in communication

Representation of cancer drugs in the media can influence peoples’ perceptions of treatment, and particular slogans or ‘buzz-words’ can be particularly consequential for patients dealing with serious illnesses. For example, ‘war’ or ‘fight’ is a common metaphor used to describe a patient’s journey through cancer [14]. This makes cancer patients feel as if the outcomes of cancer, at least in part, rely on their personal efforts; and as a result, patients subject themselves to unjustifiable toxicities in an effort to ‘win the battle’ against cancer [15]. This encourages overuse of active cancer treatments past the point of futility.

We need to do a better job of communicating the realities of cancer diagnoses to patients. For example, one group of authors recently proposed a new outcome measure of ‘days spent at home in the last 6 months of life’ to gauge the effectiveness of an investigational therapy [16]. Should we go a step further and consider whether regulatory agencies should approve drugs that provide a statistically significant OS benefit in a population of patients with an OS (not benefit) of 6 months or less?

## Are we negotiating goals of treatment effectively?

The ultimate goal of a medical intervention is to improve the duration or some measure of quality of life. Active interventions may be associated with a better sense of accomplishment for physicians, and perhaps for patients too, so we can fail to recognise that inactions may sometimes yield better outcomes than actions. When cure is not possible, prognosis is guarded severely, and treatments are accompanied by substantial trade-offs, conversations about the goals of treatment with the patients become paramount. Patients usually have an unrealistic perception of their disease stage; one survey found that only a small minority of patients in the terminal stage of their disease is aware of their prognosis [17]. By contrast, patients who discuss the prognosis of their disease with oncologists come to have a better understanding of the terminal nature of their illness [17]. The oncology community should not accept that a substantial proportion of patients have a survival expectation different from their oncologist’s [18]. Educating patients with respect to what is reasonable to hope for including any uncertainties, discussing potential toxicities of treatment and accommodating cultural and personal priorities will have crucial roles in refining patients’ goals from treatment.

## Solution

The implications of the decision to treat a terminally ill patient are multidimensional, but the ultimate goal should be to help patients with a peaceful life-death transition. To that end, education and training of oncologists on end of life care, managing expectations of patients and communication skills are important, as is the role of the media in promoting the importance and complexities of navigating quality end of life care and discouraging the ‘war’ metaphor for cancer. It is also the case that prescribing drugs in the last 6 months of life constitutes a substantial proportion of total healthcare costs, and we may be able to avoid some of these costs if patients better understand the benefit/risk trade-off offered by anticancer drugs tested in this population [19].

## Figures and Tables

**Table 1.** The survival gains, toxicity profiles and cost of three anticancer drugs with an OS of 6.5 months or less.

Drug	Study name	Year of FDA approval	Indication	Survival with the drug	Survival in control arm (control agent)	Gain in survival	SAE (drug)	SAE (control)	FAE (drug)	FAE (control)	Cost of 1 month treatment (WAC)	Reference (Pubmed ID)
Regorafenib	CORRECT	2012	Last line treatment of metastatic colorectal cancer	6.4	5.0 (placebo)	1.4	219/500 (43.8%)	100/253 (39.5%)	8/500 (1.6%)	3/253 (1.2%)	$9919	23177514
Ramucirumab	REGARD	2014	Second line treatment of metastatic gastric or gastro-oesophageal junction cancer	5.2	3.8 (placebo)	1.4	NR	NR	5/236 (2.1%)	2/115 (1.7%)	$13,093	24094768
Liposomal Irinotecan*	NAPOLI-1	2015	Second line treatment of metastatic pancreatic cancer	6.1	4.2 (fluorouracil + folinic acid)	1.9	146/264 (55.3%)	60/134 (44.8%)	5/264 (1.9%)	0/134 (0%)	$9720	26615328

Common adverse events (with incidence >10% in clinical trials) with the three drugs that provide a survival of 6 months (survival benefit of <2months).

1. Regorafenib: Fatigue, hand–foot skin reaction, diarrhoea, anorexia, hypertension, oral mucositis, rashes, nausea, weight loss, fever, thrombocytopenia.

2. Ramucirumab: Fatigue, abdominal pain, loss of appetite, vomiting, constipation, anaemia, dysphagia.

3. Nanoliposomal Irinotecan: Diarrhoea, vomiting, nausea, loss of appetite, fatigue, neutropenia, aneamia, hypokalaemia

* there were three arms in NAPOLI-1 trial: liposomal irionotecan alone (1), liposomal irinotecan +fluorouracil +folinic acid (2) and fluorouracil +folinic acid (3). Only arm 2 was shown superior and survival data corresponds to comparison between arms 2 and 3. For safety data, arms 1 and 2 are combined

WAC = wholesale acquisition costs. These were obtained from the list available at https://www.mskcc.org/sites/default/files/node/25097/documents/120915-drug-costs-table.pdf

For liposomal irinotecan, the WAC was calculated from the information available at: http://secure.medicalletter.org/w1496e

NR = not reported
